# Network Pharmacology-Guided Identification of Orientin from *Passiflora incarnata* Reveals Neuroprotective Effects Against Glutamate-Induced Excitotoxicity Through Regulation of Calcium Homeostasis

**DOI:** 10.3390/ijms27198605

**Published:** 2026-09-25

**Authors:** Jinyoung Park, Muhammad Yasir, Eun-Taek Han, Jin-Hee Han, Won Sun Park, Jongseon Choe, Wanjoo Chun

**Affiliations:** 1 Department of Pharmacology, Kangwon National University School of Medicine, Chuncheon 24341, Republic of Korea; jinyoung0326@kangwon.ac.kr (J.P.); yasir.khokhar1999@gmail.com (M.Y.); 2 Department of Medical Environmental Biology and Tropical Medicine, Kangwon National University School of Medicine, Chuncheon 24341, Republic of Korea; ethan@kangwon.ac.kr (E.-T.H.); han.han@kangwon.ac.kr (J.-H.H.); 3 Department of Physiology, Kangwon National University School of Medicine, Chuncheon 24341, Republic of Korea; parkws@kangwon.ac.kr; 4 Department of Microbiology and Immunology, Kangwon National University School of Medicine, Chuncheon 24341, Republic of Korea; jchoe@kangwon.ac.kr

**Keywords:** *Passiflora incarnata*, orientin, luteolin, network pharmacology, epilepsy, glutamate excitotoxicity, calcium homeostasis

## Abstract

Epilepsy is a chronic neurological disorder in which current antiseizure medications are limited by incomplete seizure control and variable therapeutic responses, highlighting the need to identify new bioactive candidates. *Passiflora incarnata* (*P. incarnata*) has demonstrated neuropharmacological and anticonvulsant properties. However, the individual phytochemicals and molecular mechanisms underlying these effects remain incompletely characterized. In this study, we integrated network pharmacology with cellular validation to identify epilepsy-relevant phytochemicals of *P. incarnata* and evaluate their neuroprotective effects. Eleven phytochemicals underwent target prediction and were compared with epilepsy-associated genes, yielding 147 common targets. Protein–protein interaction analysis identified 134 interacting proteins and 590 interactions, with GABA_A_ receptor subunits prominently represented among the top hub genes. Gene Ontology and Kyoto Encyclopedia of Genes and Genomes enrichment analyses further identified GABA-related functions and neuroactive ligand–receptor signaling. Integration of compound–target functional profiling with structural analysis prioritized the luteolin-derived flavonoid lineage, leading to the selection of orientin, a C-glycosylflavone constituent of *P. incarnata*, and luteolin, its structurally related core flavone scaffold, for experimental validation. In differentiated SH-SY5Y cells, pretreatment with orientin or luteolin attenuated L-glutamic acid-induced cellular injury in the presence of glycine, as evidenced by improved cell viability and reduced lactate dehydrogenase release. Both compounds also markedly reduced glutamate-induced intracellular Ca^2+^ accumulation. Notably, thapsigargin substantially reversed these Ca^2+^-lowering effects, suggesting a potential role for ER-associated Ca^2+^ regulatory mechanisms. The predicted GABAergic targets were not directly validated, and the cellular findings demonstrate neuroprotection against glutamate-induced excitotoxicity rather than direct GABA_A_ receptor modulation or antiseizure efficacy. Collectively, these findings demonstrate the utility of network pharmacology-guided prioritization for identifying bioactive constituents of *P. incarnata* and support orientin as a neuroprotective candidate for further mechanistic and in vivo investigation.

## 1. Introduction

Epilepsy is a chronic neurological disorder characterized by recurrent seizures resulting from abnormal and excessive neuronal activity. Antiseizure medications (ASMs) remain the primary pharmacological approach for seizure control and act through diverse mechanisms, including modulation of voltage-gated ion channels, enhancement of GABAergic inhibition, and attenuation of glutamatergic excitation [1]. Despite the availability of multiple ASMs with distinct mechanisms of action, approximately one-third of patients fail to achieve adequate seizure control [2,3], and long-term treatment is frequently complicated by adverse effects, drug–drug interactions, and variable therapeutic responses [4,5,6,7,8,9,10,11]. Moreover, current ASMs are primarily used for symptomatic seizure control, while their ability to modify the underlying processes of epileptogenesis remains limited [1,12,13]. These limitations highlight the continuing need to identify new bioactive compounds with distinct chemical structures and pharmacological properties. In this context, natural products represent a valuable source of structurally diverse and biologically active molecules and have historically contributed to the discovery and development of therapeutically important agents [14]. Their chemical diversity and capacity to modulate multiple molecular targets make natural products particularly attractive as a source of candidate compounds for the development of new therapeutic strategies for epilepsy [11].

Among medicinal plants with potential neurological activities, *Passiflora incarnata* L. (Passifloraceae), commonly known as passionflower, has attracted attention because of its traditional use for the treatment of anxiety, insomnia, and other nervous disorders [15]. Experimental studies have further demonstrated neuropharmacological properties of *P. incarnata*, including anxiolytic, sedative, and anticonvulsant activities [16]. In particular, *P. incarnata* extracts have been reported to modulate GABAergic neurotransmission through effects on GABA uptake and interaction with GABA receptors, and anticonvulsant effects have been observed in experimental seizure models [17,18,19,20]. These findings suggest that *P. incarnata* contains bioactive constituents capable of modulating molecular processes relevant to neuronal excitability and seizure susceptibility. However, *P. incarnata* contains structurally diverse phytochemicals, including flavonoids and β-carboline alkaloids, and most previous studies have evaluated crude extracts or fractions rather than systematically identifying the individual constituents responsible for the epilepsy-related pharmacological effects of *P. incarnata* [15,21,22,23,24]. Consequently, the specific phytochemicals, molecular targets, and biological pathways underlying the potential anticonvulsant and neuroprotective properties of *P. incarnata* remain incompletely understood [25,26].

Network pharmacology provides a systematic approach for investigating the complex pharmacological actions of medicinal plants by integrating multiple compounds, molecular targets, and disease-associated pathways within a unified network framework [22,27]. This approach is particularly suitable for *P. incarnata*, in which structurally diverse phytochemicals may interact with multiple, functionally interconnected targets. By combining phytochemical target prediction with disease-associated genes, protein–protein interaction (PPI) analysis, and functional enrichment analysis, network pharmacology can facilitate the identification of molecular networks potentially relevant to epilepsy and enable the prioritization of candidate compounds for further investigation [28,29]. However, computational predictions alone cannot establish biological activity or therapeutic relevance. Experimental validation of network-prioritized compounds is therefore essential to determine whether the prioritized compounds exert biologically relevant effects in neuronal systems [30,31].

Glutamate-mediated excitotoxicity is an important mechanism of neuronal injury associated with epilepsy [32,33,34,35,36], in which excessive excitatory signaling can disrupt intracellular Ca^2+^ homeostasis and promote neuronal dysfunction and cell death [37,38]. Sustained elevation of cytosolic Ca^2+^ can trigger multiple downstream events, including oxidative stress, mitochondrial dysfunction, and activation of Ca^2+^-dependent cell-death pathways [39,40,41,42]. Thus, glutamate-induced neuronal injury and intracellular Ca^2+^ dysregulation provide experimentally tractable cellular endpoints for investigating an epilepsy-relevant mechanism of neuronal injury [43]. Nevertheless, glutamate-induced excitotoxicity in a cultured neuronal cell model represents only a simplified simulation of one component of the complex pathophysiology of epilepsy and does not reproduce the neuronal network hyperexcitability, recurrent seizure activity, or excitatory–inhibitory circuit interactions that characterize the disease in vivo. Accordingly, cellular protection against glutamate-induced excitotoxicity should be interpreted as evidence of activity against an epilepsy-relevant neuronal injury mechanism rather than as direct evidence of anti-epileptic efficacy.

In the present study, we integrated network pharmacology with functional cellular validation to systematically investigate the epilepsy-associated molecular network of *P. incarnata* phytochemicals and to identify candidate compounds for experimental evaluation. Compound–target relationships, functional enrichment profiles, and structural characteristics were integrated to guide candidate selection. The neuroprotective effects of the selected compounds, orientin and luteolin, were subsequently evaluated in all-trans retinoic acid (ATRA)-differentiated SH-SY5Y cells, which exhibit neuron-like characteristics but do not constitute bona fide mature neurons or reproduce an intact epileptic neuronal network, exposed to glutamate-induced excitotoxic stress, with particular emphasis on intracellular Ca^2+^ homeostasis. Accordingly, the cellular experiments were designed to provide mechanistic validation of neuroprotective activity against epilepsy-relevant excitotoxic stress rather than to demonstrate direct anti-epileptic activity. By linking network-based phytochemical prioritization with experimental assessment of neuronal injury and Ca^2+^ responses, this study provides a systematic framework for identifying neuroprotective constituents of *P. incarnata* and exploring their potential relevance to epilepsy-associated excitotoxic mechanisms.

## 2. Result

### 2.1. Identification of Potential Therapeutic Targets of P. incarnata Against Epilepsy

A total of 210 target genes associated with the phytochemical constituents of *P. incarnata* were identified through target prediction analysis. In addition, 10,045 epilepsy-related genes were retrieved from the GeneCards database. To identify the potential therapeutic targets of *P. incarnata* against epilepsy, the two datasets were integrated using Venn diagram analysis. As shown in Figure 1, 147 overlapping target genes were identified, representing the common targets between *P. incarnata* and epilepsy. These common targets were subsequently subjected to protein–protein interaction (PPI) network construction and functional enrichment analyses.

### 2.2. Construction of the Protein–Protein Interaction Network

A protein–protein interaction (PPI) network was constructed to investigate the interactions among the common target genes shared between *P. incarnata* and epilepsy. After excluding 13 isolated genes that showed no interactions, the final network consisted of 134 nodes and 590 edges (Figure 2). Each node represents a target gene, whereas each edge represents a protein–protein interaction between the corresponding gene products. Based on the Degree values, the target genes exhibited varying levels of connectivity, with highly connected hub genes displayed in red and genes with lower connectivity displayed in yellow.

### 2.3. Identification of Hub Genes

To identify key genes within the PPI network, hub genes were ranked using the Maximal Clique Centrality (MCC) algorithm in cytoHubba. The top 20 genes ranked by MCC score are presented in Table 1. CCND1 exhibited the highest MCC score (13,332), followed by CYP3A4, CYP1A2, NR1I2, and CYP1A1. Notably, several GABA receptor-related genes, including GABRA1, GABRG2, GABRA4, GABRA5, GABRA3, GABRA6, and GABRA2, were also identified among the top 20 hub genes.

### 2.4. Functional Enrichment Analysis of Common Target Genes

Gene Ontology (GO) enrichment analysis was performed to investigate the biological functions of the 147 common target genes shared between *P. incarnata* and epilepsy. The top 10 enriched terms for Biological Process (BP), Cellular Component (CC), and Molecular Function (MF) are presented in Figure 3A. In the BP category, the enriched terms were primarily associated with protein phosphorylation, gamma-aminobutyric acid (GABA) signaling, synaptic signaling, chemical synaptic transmission, and the regulation of transmembrane transport, suggesting the involvement of the common target genes in neuronal signaling and synaptic function. The CC category showed significant enrichment in the postsynaptic specialization membrane, GABA receptor complex, chloride channel complex, and synapse, whereas the MF category was mainly enriched in kinase activity, protein kinase activity, GABA-gated chloride ion channel activity, ATP binding, and GABA_A_ receptor activity.

KEGG pathway enrichment analysis identified several significantly enriched pathways (Figure 3B). Among these, Neuroactive ligand–receptor interaction exhibited the highest enrichment, followed by pathways related to serotonergic synapse, calcium signaling pathway, and nicotine addiction. These enriched pathways suggest that the common target genes are primarily involved in neurotransmission, synaptic signaling, and neuronal excitability, which are closely associated with the pathophysiology of epilepsy.

### 2.5. Active Compound–Target Relationships and Functional Profiles

To further characterize the relationships between the phytochemical constituents of *P. incarnata* and key targets associated with epilepsy, an active compound–target–epilepsy network was constructed using the top 20 hub genes identified by MCC analysis (Figure 4A). The network revealed extensive compound–target connectivity, with individual phytochemicals predicted to interact with multiple hub genes and several hub genes shared by multiple compounds. The major hub targets included genes involved in cell-cycle regulation (CCND1, CCNA2, CCNB1, CDK2, and CDKN1A), xenobiotic metabolism and transport (CYP3A4, CYP1A2, CYP1A1, CYP1B1, ABCB1, and ABCG2), and GABAergic neurotransmission. Notably, seven GABA_A_ receptor subunit genes (GABRA1–6 and GABRG2) were included among the top 20 MCC-ranked hub genes. Consistent with this finding, the preceding enrichment analyses identified neuroactive ligand signaling and neuroactive ligand–receptor interaction among the significantly enriched KEGG pathways, together with multiple GABA-related terms in the GO analysis. Collectively, these findings identified GABAergic and neuroactive receptor signaling as prominent network-level features linking *P. incarnata* phytochemicals to epilepsy-associated targets.

To further characterize functionally relevant compound–target relationships, predicted associations involving targets derived from significantly enriched neuronal GO terms were visualized as a binary compound–target matrix (Figure 4B). The selected targets were classified into four functional categories: GABAergic neurotransmission, monoaminergic neurotransmission, synaptic excitability and neuronal regulation, and carbonic anhydrase/pH regulation. Distinct target-association patterns were observed among the phytochemical constituents. The β-carboline alkaloids, particularly harman and norharman, showed prominent associations with multiple GABA_A_ receptor subunits, whereas several other β-carbolines were associated with monoaminergic targets. In contrast, the flavonoid constituents, including orientin, meloside L, meloside A, and saponarin, exhibited predicted associations with targets involved in neuronal regulation and excitability, as well as with multiple carbonic anhydrase isoforms. These findings indicate that the predicted target profiles of *P. incarnata* phytochemicals extend beyond the prominent GABAergic hub signature and involve multiple functional systems relevant to neuronal signaling and excitability.

The functional target profiles were further considered together with the structural characteristics of the phytochemical constituents. Structural comparison showed that orientin and meloside L belong to the luteolin-derived C-glycosylflavone lineage (Figure 4C). Orientin is luteolin-8-C-glucoside, whereas meloside L is a diglycosylated luteolin derivative based on the isoorientin (luteolin-6-C-glucoside) scaffold. In contrast, meloside A and saponarin belong to the structurally distinct apigenin-derived flavone lineage, whereas most of the remaining compounds are β-carboline alkaloids. Based on the integration of the predicted compound–target profiles, GO-derived neuronal relevance, and structural relationships, the luteolin-derived flavonoid lineage was selected for subsequent experimental investigation. Orientin was selected as a representative C-glycosylflavone constituent of *P. incarnata*, whereas luteolin was selected as the core flavone scaffold of the luteolin-derived candidates orientin and meloside L. Accordingly, orientin and luteolin were selected for subsequent experimental validation using glutamate-induced excitotoxicity and intracellular Ca^2+^ imaging assays.

### 2.6. Protective Effects of Orientin and Luteolin Against Glutamate-Induced Excitotoxicity

To determine an appropriate concentration of L-glutamic acid for inducing excitotoxic injury in differentiated SH-SY5Y cells, cells were treated with various concentrations of L-glutamic acid in the presence of 10 μM glycine, and cytotoxicity was evaluated using a lactate dehydrogenase (LDH) assay. LDH release markedly increased at higher concentrations of L-glutamic acid, with a pronounced cytotoxic response observed at 50 and 100 μM (Figure 5A). Based on these results, 100 μM L-glutamic acid in combination with 10 μM glycine was selected for subsequent experiments. For clarity, this treatment condition is hereafter referred to as glutamate treatment, and the resulting cellular injury as glutamate-induced excitotoxicity.

The protective effect of orientin against glutamate-induced excitotoxicity was subsequently evaluated using Cell Counting Kit-8 (CCK-8) and LDH assays (Figure 5B). Glutamate treatment markedly reduced cell viability and increased LDH release compared with the control group. Pretreatment with orientin significantly improved cell viability, with the greatest recovery observed at 5 μM. Consistently, orientin significantly reduced glutamate-induced LDH release across the tested concentrations, indicating attenuation of glutamate-induced cellular injury. Luteolin also exerted significant protective effects against glutamate-induced excitotoxicity (Figure 5C). Pretreatment with luteolin at 0.5–10 μM significantly increased cell viability compared with the glutamate-treated group, with a pronounced recovery observed at 1 μM.

Consistently, luteolin significantly reduced glutamate-induced LDH release across the tested concentrations. Together, these findings demonstrate that both orientin and luteolin attenuated glutamate-induced excitotoxic injury in differentiated SH-SY5Y cells, as evidenced by improved cell viability and reduced LDH release.

### 2.7. Effect of Orientin and Luteolin on Intracellular Ca^2+^ Levels

To investigate whether the protective effects of orientin and luteolin against glutamate-induced excitotoxicity were associated with the regulation of intracellular Ca^2+^ levels, Fluo-4 AM-based Ca^2+^ imaging was performed in differentiated SH-SY5Y cells. Representative fluorescence images showed that treatment with 100 μM L-glutamic acid in the presence of 10 μM glycine markedly increased Fluo-4 fluorescence compared with the control group, whereas pretreatment with either orientin or luteolin substantially reduced the glutamate-induced fluorescence signal (Figure 6A).

Quantitative analysis of Fluo-4 fluorescence intensity confirmed these observations (Figure 6B). Glutamate treatment significantly increased intracellular Ca^2+^-dependent fluorescence compared with the control group, whereas pretreatment with either orientin or luteolin significantly reduced the glutamate-induced increase (*p* < 0.001). To further investigate the potential involvement of endoplasmic reticulum (ER)-associated Ca^2+^ regulatory mechanisms, cells were treated with thapsigargin, an inhibitor of sarco/endoplasmic reticulum Ca^2+^-ATPase (SERCA). Thapsigargin significantly reversed the Ca^2+^-lowering effects of both orientin and luteolin, as evidenced by increased Fluo-4 fluorescence compared with the respective glutamate–orientin and glutamate–luteolin groups (*p* < 0.001). In the absence of glutamate, Fluo-4 fluorescence was significantly higher in cells treated with orientin or luteolin in combination with thapsigargin than in cells treated with thapsigargin alone (*p* < 0.001; Supplementary Figure S1).

Collectively, these findings demonstrate that orientin and luteolin attenuated glutamate-induced intracellular Ca^2+^ elevation in differentiated SH-SY5Y cells and that thapsigargin diminished these Ca^2+^-lowering effects, suggesting a potential role for ER-associated mechanisms in intracellular Ca^2+^ homeostasis.

## 3. Discussion

Despite the availability of multiple antiseizure medications (ASMs) with diverse mechanisms of action [1], a substantial proportion of patients with epilepsy do not achieve adequate seizure control, and long-term pharmacotherapy can be limited by adverse effects, drug–drug interactions, and variable therapeutic responses [18,19]. These limitations support continued efforts to identify bioactive compounds with distinct chemical and pharmacological properties. *P. incarnata* has attracted attention in this context because its extracts have shown anticonvulsant activity in experimental seizure models and have been reported to affect GABAergic neurotransmission. However, most previous studies have examined extracts or fractions containing multiple constituents, making it difficult to determine which individual phytochemicals contribute to the observed effects and which molecular targets are associated with their activity. The present study was therefore designed to identify epilepsy-relevant phytochemicals of *P. incarnata* through network pharmacology and to experimentally evaluate the selected candidates in a neuronal model of excitotoxic injury.

Network analysis provided a systematic basis for examining the relationship between *P. incarnata* phytochemicals and epilepsy-associated molecular targets. Among the predicted compound targets and epilepsy-associated genes, 147 common targets were identified, and subsequent PPI analysis identified highly connected hub proteins. Notably, seven GABA_A_ receptor subunits (GABRA1–6 and GABRG2) were included among the top 20 MCC-ranked hub genes. GO and KEGG enrichment analyses further identified GABA-related functions and neuroactive ligand–receptor interaction, supporting the neurological relevance of the common-target network. These findings are consistent with previous reports of GABA-related pharmacological effects of *P. incarnata* extracts and provide a network-level context for their reported anticonvulsant activity [17]. Nevertheless, the PPI and enrichment results represent associations derived from predicted targets and should not be interpreted as evidence that individual *P. incarnata* phytochemicals directly interact with these GABAergic targets.

Because hub-gene ranking of the integrated network does not by itself identify the most relevant compound for experimental validation [22], compound-specific target relationships were further evaluated using GO-derived functional categories related to neuronal signaling and excitability (Figure 4B). The resulting matrix showed distinct target profiles among the 11 phytochemicals across GABAergic neurotransmission, monoaminergic neurotransmission, synaptic excitability/neuronal regulation, and carbonic anhydrase/pH regulation. These functional relationships were considered together with structural classification of the phytochemicals. Orientin and meloside L were identified as members of a luteolin-derived C-glycosylflavone lineage, whereas meloside A and saponarin were associated with an apigenin-derived flavone lineage and most of the remaining constituents were β-carboline alkaloids. Although several β-carboline alkaloids showed prominent associations with GABAergic targets, network connectivity was not considered equivalent to suitability for further drug development, and safety considerations were also incorporated into candidate prioritization. Previous studies have reported neurotoxic effects associated with β-carbolines relevant to the present network, including harman, norharman, and harmine [44,45], raising concerns regarding their prioritization as neuroprotective candidates. Accordingly, these β-carbolines were not prioritized for subsequent cellular validation despite their prominent predicted associations with GABAergic targets. Structural comparison further showed orientin as luteolin-8-C-glucoside and meloside L as a more extensively glycosylated luteolin derivative (Figure 4C). Given these considerations, orientin was selected as a representative C-glycosylflavone constituent of *P. incarnata*, while luteolin was evaluated as the core flavone scaffold of the luteolin-derived candidates. Thus, the subsequent cellular experiments were designed as a phenotype-based evaluation of the prioritized flavonoid candidates rather than as direct validation of the predicted GABAergic hub targets.

This strategy enabled candidate selection to be based on network-derived biological relevance, structural relationships, and safety considerations rather than on overall hub-gene ranking alone.

Experimental validation demonstrated that pretreatment with orientin or luteolin attenuated cellular injury induced by L-glutamic acid in the presence of glycine in differentiated SH-SY5Y cells, as evidenced by improved cell viability and reduced LDH release. The protective effect of luteolin observed in the present study is consistent with previous reports demonstrating its neuroprotective activity against glutamate-induced neuronal injury [46]. Intracellular Ca^2+^ imaging further showed that the glutamate-induced elevation of cytosolic Ca^2+^ was markedly attenuated by pretreatment with either orientin or luteolin. Consistent with our findings, orientin has previously been reported to reduce intracellular Ca^2+^ accumulation and cellular injury in primary cortical neurons exposed to oxygen–glucose deprivation/reperfusion [47]. Excessive cytosolic Ca^2+^ accumulation is an important component of glutamate-mediated excitotoxicity and contributes to neuronal injury through Ca^2+^-dependent cellular processes [32,33]. Taken together, the parallel attenuation of cellular injury and intracellular Ca^2+^ accumulation suggests that preservation of intracellular Ca^2+^ homeostasis may contribute to the neuroprotective effects of orientin and luteolin. To further investigate the potential contribution of intracellular Ca^2+^ stores, thapsigargin was used to pharmacologically disrupt ER Ca^2+^ homeostasis through inhibition of SERCA. The ER is a major intracellular Ca^2+^ store, and SERCA plays a critical role in maintaining ER Ca^2+^ homeostasis by transporting cytosolic Ca^2+^ into the ER [22,23,24]. In the present study, thapsigargin substantially attenuated the Ca^2+^-lowering effects of orientin and luteolin under glutamate-challenged conditions, resulting in a significant recovery of cytosolic Ca^2+^ signals. This pharmacological response indicates that the Ca^2+^-lowering effects of orientin and luteolin contain a substantial thapsigargin-sensitive component and suggests a potential contribution of ER-associated Ca^2+^ regulatory mechanisms to intracellular Ca^2+^ homeostasis.

Nevertheless, these findings should not be interpreted as evidence that orientin or luteolin directly enhance SERCA activity, modulate inositol 1,4,5-trisphosphate receptors (IP3Rs), or promote Ca^2+^ sequestration into the ER. In addition to depleting ER Ca^2+^ stores through SERCA inhibition, thapsigargin can induce ER stress and consequently influence multiple Ca^2+^-dependent signaling pathways. Moreover, alternative mechanisms regulating cytosolic Ca^2+^, including plasma membrane Ca^2+^ transport, Na^+^/Ca^2+^ exchange, and mitochondrial Ca^2+^ uptake, may also contribute to the effects of orientin and luteolin. Because ER luminal Ca^2+^ content and SERCA activity were not directly measured in the present study, the specific molecular mechanism underlying the observed thapsigargin-sensitive Ca^2+^ regulation remains to be determined. Further studies directly assessing ER Ca^2+^ dynamics, SERCA activity, and other Ca^2+^-regulatory pathways will therefore be required to define the underlying mechanism.

The present study also has limitations regarding the experimental model used to assess the epilepsy-related relevance of the network pharmacology findings. Although ATRA-differentiated SH-SY5Y cells exhibit neuron-like characteristics and are widely used to investigate neurotoxicity and neuroprotective mechanisms, they remain neuroblastoma-derived cells and do not reproduce the mature neuronal circuitry, excitatory–inhibitory network interactions, or recurrent epileptiform activity characteristic of epilepsy. Furthermore, glutamate-induced excitotoxicity represents only one component of the complex and multifactorial pathophysiology of epilepsy. Therefore, the observed reductions in glutamate-induced cellular injury and intracellular Ca^2+^ accumulation should be interpreted as evidence of neuroprotective activity against an epilepsy-relevant excitotoxic mechanism rather than as direct evidence of anti-epileptic efficacy. Confirmation of these findings in primary neuronal cultures or human induced pluripotent stem cell (iPSC)-derived neurons would strengthen their physiological relevance, while electrophysiological studies and established in vivo epilepsy models will be required to determine whether orientin and luteolin can modulate neuronal hyperexcitability and seizure activity and thereby establish their pharmacodynamic relevance to epilepsy.

## 4. Materials and Methods

### 4.1. Mining of Active Compounds and Targets in Passiflora incarnata

The phytochemical constituents of *P. incarnata* were collected from the KNApSAcK Family database (https://www.knapsackfamily.com/KNApSAcK/) (accessed on 11 September 2025). The pharmacokinetic properties of the identified compounds, including drug-likeness and oral bioavailability, were evaluated using the SwissADME web server (http://www.swissadme.ch) (accessed on 4 March 2026). Subsequently, all identified compounds were subjected to target prediction using the SwissTargetPrediction database (http://www.swisstargetprediction.ch) (accessed on 2 February 2026), with the organism restricted to *Homo sapiens*. Only predicted targets with a probability >0.001 were retained for subsequent analysis, whereas predictions with a probability ≤0.001 were excluded. The retained target names were standardized to official gene symbols using UniProt, and duplicate targets were removed to generate a non-redundant set of compound-associated targets. The targets related to epilepsy were obtained from the GeneCards database (https://www.genecards.org/) (accessed on 2 February 2026). The overlapping targets between *P. incarnata* and epilepsy were identified using Draw Venn Diagram (http://bioinformatics.psb.ugent.be/webtools/Venn/ (accessed on 9 February 2026)).

### 4.2. Protein–Protein Interaction Network Construction and Analysis

The common targets between *P. incarnata* and epilepsy were imported into the STRING database (version 12.0; http://string-db.org/; accessed on 10 February 2026) to construct a protein–protein interaction (PPI) network. The species was restricted to *Homo sapiens*, the minimum required interaction score was set to medium confidence (0.400), while all other parameters were maintained at their default settings. The resulting PPI network was imported into Cytoscape (version 3.10.1) for network visualization and topological analysis.

The degree algorithm in cytoHubba plugin was used to assess node connectivity and visualize the PPI network, with node colors reflecting their degree values. In addition, the Maximal Clique Centrality (MCC) algorithm was applied to identify and rank hub genes within the network. The top 20 genes ranked by MCC score were identified as the major hub genes within the PPI network.

### 4.3. GO and KEGG Enrichment Analysis

The common genes shared between *P. incarnata* and epilepsy were subjected to Gene Ontology (GO) functional annotation and Kyoto Encyclopedia of Genes and Genomes (KEGG) pathway enrichment analyses using the Database for Annotation, Visualization and Integrated Discovery (DAVID, Knowledgebase v2025_2; https://davidbioinformatics.nih.gov/tools.jsp; accessed on 23 February 2026). The gene identifier was set to ‘OFFICIAL_GENE_SYMBOL’, and the species was restricted to *Homo sapiens*.

GO enrichment analysis was performed to identify significantly enriched Biological Processes (BP), Molecular Function (MF), and Cellular Components (CC), while KEGG pathway enrichment analysis was conducted to identify the biological pathways potentially involved in the therapeutic effects of *P. incarnata* and epilepsy. Enrichment results with a *p*-value < 0.05 were considered statistically significant. The top 10 enriched GO terms and KEGG pathways, ranked according to the *p*-value, were selected for visualization using the SRPLOT platform (http://www.bioinformatics.com.cn/en; accessed on 23 Febtuary 2026).

To facilitate biological interpretation, the significantly enriched KEGG pathways were further classified into functional categories based on their biological relevance to epilepsy.

### 4.4. Construction and Functional Characterization of the Active Compound–Target–Epilepsy Network

An active compound–target–epilepsy network was constructed to visualize the relationships among the active compounds of Passiflora incarnata, their predicted target genes, and epilepsy. The top 20 hub genes identified by the MCC algorithm were used to construct the network. The network was visualized as a Sankey diagram using SankeyMATIC (http://sankeymatic.com/build/, accessed on 25 February 2026), in which the links represent the predicted associations between active compounds, their corresponding hub genes, and epilepsy.

To further characterize functionally relevant compound–target associations, significantly enriched GO terms (*p* < 0.05) directly related to neuronal signaling and excitability were categorized according to their biological functions, including GABAergic neurotransmission, monoaminergic neurotransmission, synaptic excitability and neuronal regulation, and carbonic anhydrase-mediated pH regulation. Genes annotated to these functional terms were retained if they were predicted as targets of at least one *P. incarnata* phytochemical. Compound–target associations obtained from SwissTargetPrediction were visualized as a binary matrix according to the GO-defined functional categories.

### 4.5. Experimental Reagent and Cell Culture

Human neuroblastoma SH-SY5Y cells were obtained from the Korean Cell Line Bank (KCLB, #22266, Seoul, Republic of Korea). Cells were routinely maintained in Minimum Essential Medium (MEM; BYLABS, #BY0311) supplemented with 10% heat-inactivated fetal bovine serum (FBS; Gibco, Grand Island, NY, USA) and 100 U/mL penicillin–streptomycin (Gibco) at 37 °C in a humidified incubator containing 5% CO_2_.

For neuronal differentiation, SH-SY5Y cells were seeded and allowed to attach for 1 day. The culture medium was then replaced with DMEM/F-12 medium (Invitrogen, Carlsbad, CA, USA; #11330-032) supplemented with 1% FBS and 10 μM all-trans retinoic acid (ATRA; Sigma, St. Louis, MO, USA, #R2625). The differentiation medium was refreshed every 2 days, and the cells were differentiated for 6 days at 37 °C in a humidified incubator containing 5% CO_2_. All subsequent experiments were performed using differentiated SH-SY5Y cells.

### 4.6. Glutamate-Induced Excitotoxicity Assay

Differentiated SH-SY5Y cells were pretreated with orientin (MCE, Monnouth Juncion, NJ, USA, HY-N0405) or luteolin (MCE, Monnouth Juncion, NJ, USA, HY-N0162) for 1 day, followed by exposure to 100 μM L-glutamic acid and 10 μM glycine for an additional 1 day to induce glutamate-mediated excitotoxicity. Glycine was included as a co-agonist of the NMDA receptor to facilitate glutamate-induced NMDA receptor activation and the associated excitotoxic response [48,49]. Cell viability was evaluated using the Cell Counting Kit-8 (CCK-8; Dojindo Laboratories, Kumamoto, Japan; #CK04), and cytotoxicity was assessed using a lactate dehydrogenase (LDH) assay kit (DOGENBIO, Seoul, Republic of Korea; #DG-LDH500). Both assays were performed according to the manufacturers’ instructions.

### 4.7. Intracellular Ca^2+^ Imaging Assay

Glass coverslips (18 mm diameter) were incubated in 1 M HCl for 1 h at room temperature, rinsed five times with distilled water, and dried in a dry oven at 180 °C for 2 h. The coverslips were coated with 1 μg/mL poly-D-lysine hydrobromide (Sigma-Aldrich, St. Louis, MO, USA; #P6407) at 37 °C for 2 h, washed three times with distilled water, air-dried under sterile conditions, and subsequently coated with 10 μg/mL laminin in phosphate-buffered saline (PBS) at 37 °C for 4 h. After removal of the laminin solution, SH-SY5Y cells were immediately seeded onto the coated coverslips.

The cells were differentiated for 6 days in DMEM/F-12 medium supplemented with 1% FBS and 10 μM all-trans retinoic acid, as described above. Differentiated SH-SY5Y cells were pretreated with 10 μM orientin or 5 μM luteolin for 1 day, followed by treatment with 100 μM L-glutamic acid and 10 μM glycine for an additional 1 day.

Following treatment, the cells were incubated with 2 μM Fluo-4 AM (Invitrogen, Thermo Fisher Scientific, Waltham, MA, USA; # F14201), prepared in serum-free Hanks’ Balanced Salt Solution (HBSS; Invitrogen, Thermo Fisher Scientific, Waltham, MA, USA; # 14175), containing no calcium, no magnesium, and no phenol red, supplemented with 2 mM CaCl_2_ (final concentration), for 30 min at 37 °C. After staining, the cells were washed with HBSS, and intracellular Ca^2+^ fluorescence was acquired using a confocal laser scanning microscope (K1-fluo; Nanoscope Systems, Daejon, Republic of Korea) with excitation and emission wavelengths of 495 nm and 506 nm, respectively.

### 4.8. Statistical Analysis

All quantitative data are expressed as the mean ± standard deviation (SD). For comparisons involving multiple experimental groups, statistical significance was assessed using one-way analysis of variance (ANOVA) followed by an appropriate multiple-comparison test. Dunnett’s multiple-comparisons test was used when multiple treatment groups were compared with a single reference group. Tukey’s multiple-comparisons test was used when pairwise comparisons among multiple treatment groups were required. A *p*-value < 0.05 was considered statistically significant. Statistical significance is indicated as *p* < 0.05 (*), *p* < 0.01 (**), and *p* < 0.001 (***).

## 5. Conclusions

In the present study, network pharmacology and experimental validation were integrated to identify and evaluate epilepsy-relevant phytochemicals from *P. incarnata*. PPI and functional enrichment analyses revealed epilepsy-associated molecular networks involving GABAergic and neuroactive signaling, while compound-specific target profiling and structural comparison supported the prioritization of the luteolin-derived flavonoid lineage. Orientin was selected as a representative C-glycosylflavone constituent of *P. incarnata*, and luteolin was evaluated as its structurally related core flavone scaffold. Both compounds attenuated glutamate-induced cellular injury and intracellular Ca^2+^ accumulation in differentiated SH-SY5Y cells, and the reversal of their Ca^2+^-lowering effects by thapsigargin suggested a potential role for ER-associated Ca^2+^ regulatory mechanisms. A major strength of this study is the systematic integration of network-guided candidate prioritization with phenotype-level cellular validation, providing neuroprotective evidence against glutamate-induced excitotoxicity for the selected compounds. However, the predicted compound–target relationships, including the GABAergic hub targets identified by network analysis, require direct target-level validation, and the cellular excitotoxicity model used here represents a simplified model of epilepsy-relevant neuronal injury and does not provide evidence of direct GABA_A_ receptor modulation or antiseizure efficacy. Further studies using target-specific assays, direct assessment of ER Ca^2+^ regulation, electrophysiological approaches, and established in vivo epilepsy models are warranted to define the molecular mechanisms of orientin and luteolin and to determine whether their neuroprotective effects translate into modulation of neuronal hyperexcitability and seizure activity in vivo.

## Figures and Tables

**Figure 1:**
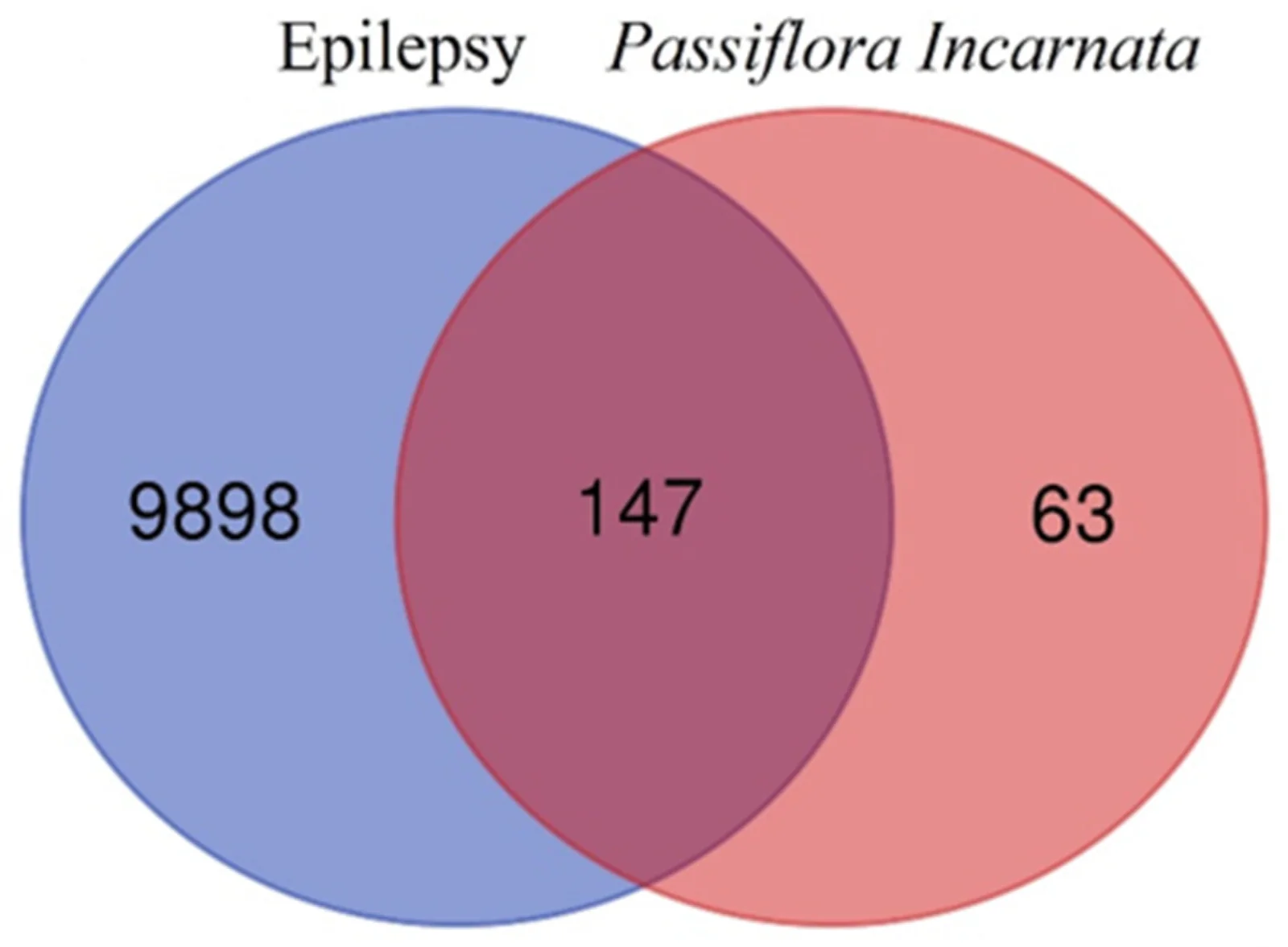
Venn diagram illustrating the overlap of target genes between *P. incarnata* and epilepsy. The blue region represents epilepsy-associated target genes, the red region represents *P. incarnata*-associated target genes, and the overlapping region represents the 147 common target genes shared between *P. incarnata* and epilepsy.

**Figure 2:**
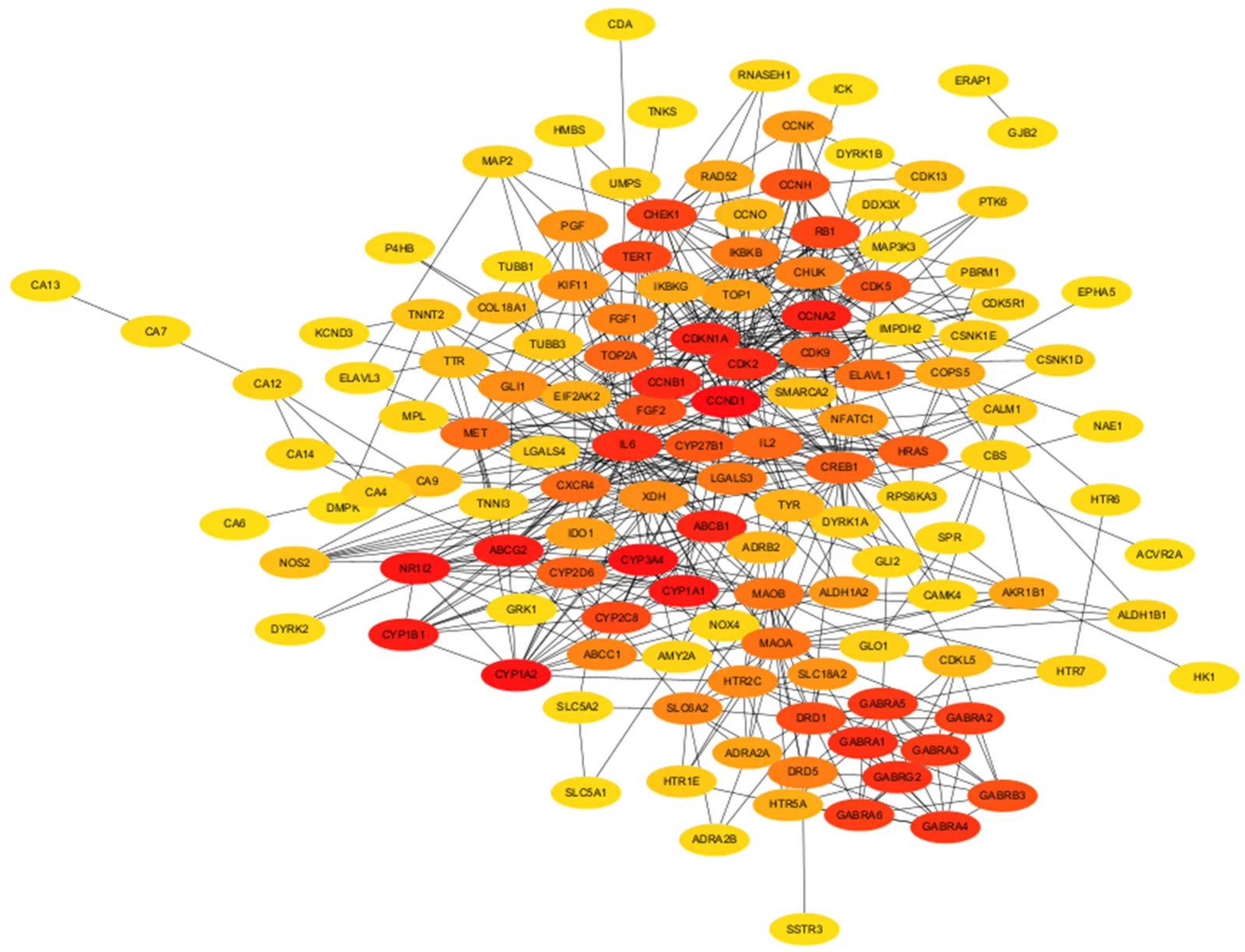
Protein–protein interaction (PPI) network of the common target genes shared between *P. incarnata* and epilepsy. The network consists of 134 nodes and 590 edges. Nodes are colored according to their degree values, from yellow (low) to red (high).

**Figure 3:**
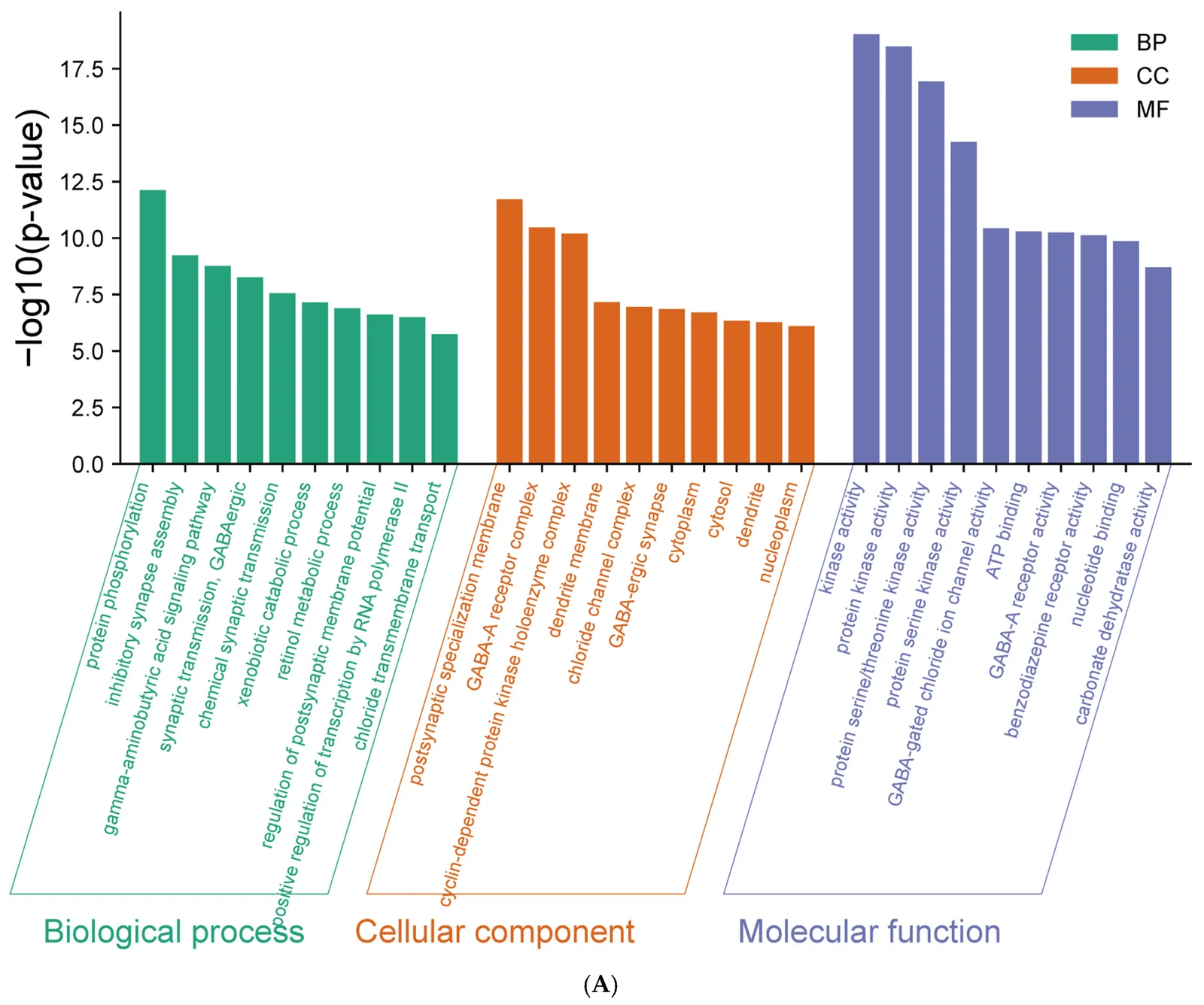

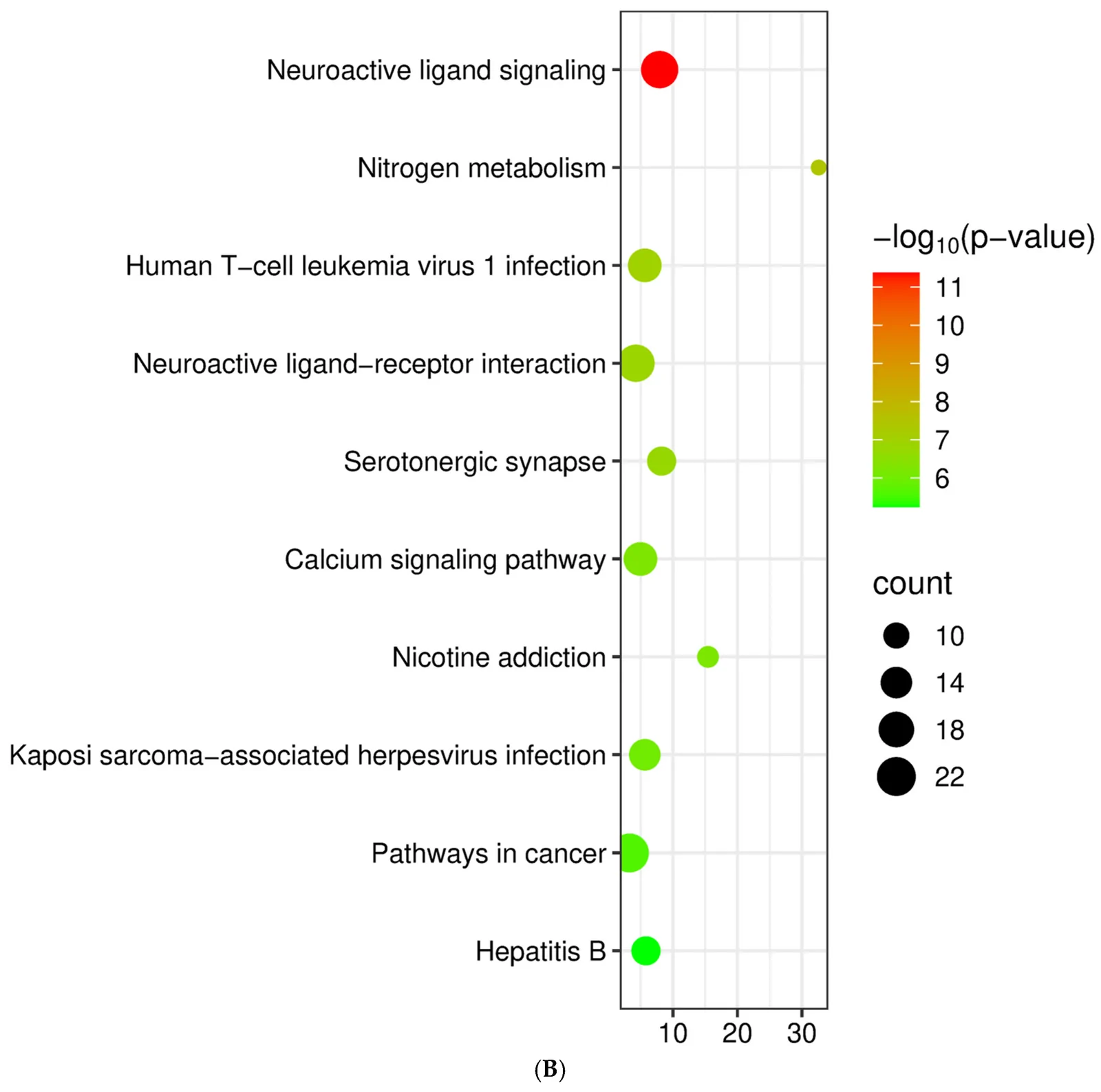
Gene Ontology (GO) and KEGG enrichment analyses of the common target genes shared between *P. incarnata* and epilepsy. (**A**) GO enrichment analysis showing the top 10 significantly enriched terms in the Biological Process (BP), Cellular Component (CC), and Molecular Function (MF) categories. (**B**) KEGG pathway enrichment analysis showing the top 10 enriched pathways. Bubble size represents the gene count, and bubble color represents the enrichment significance (−log_10_(*p*-value)).

**Figure 4:**
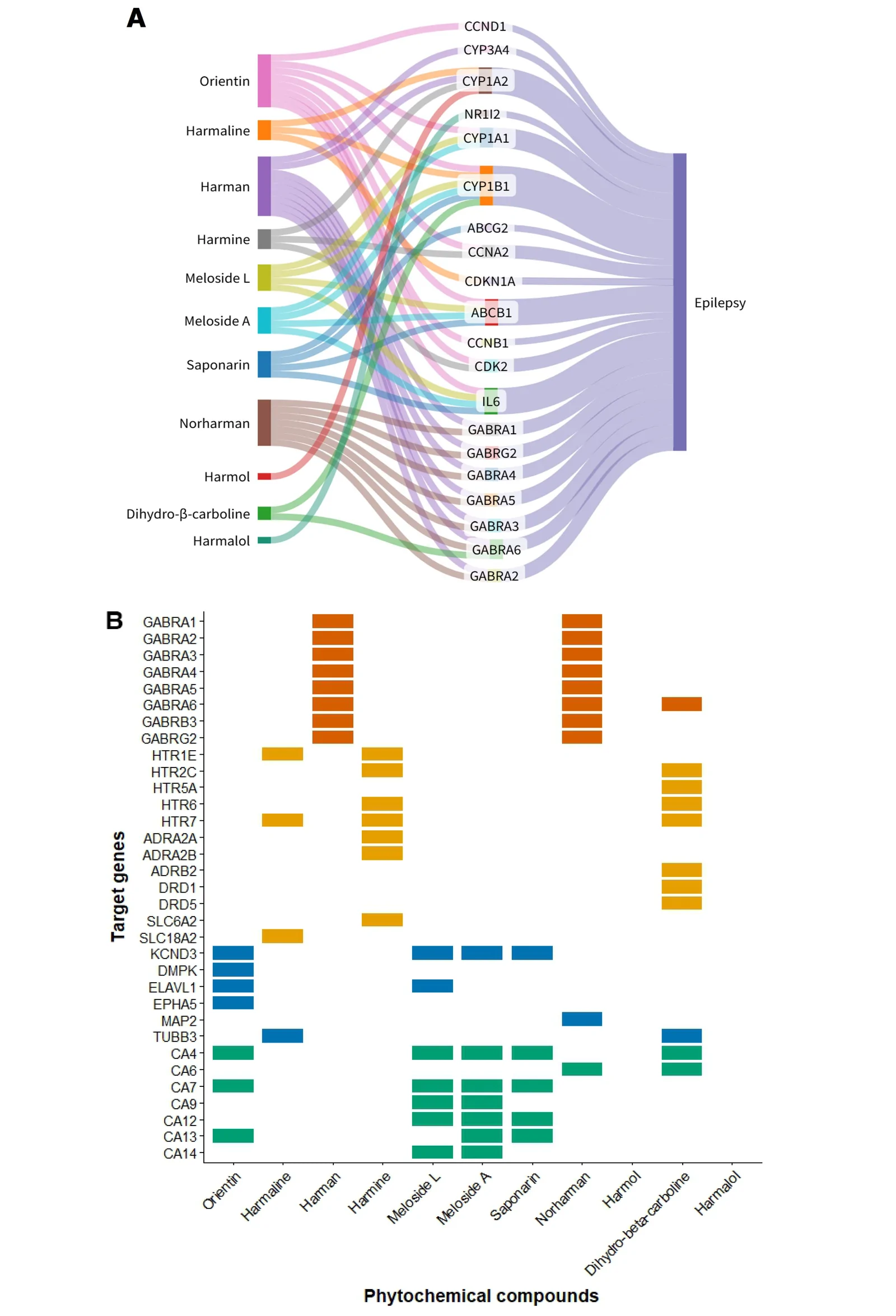

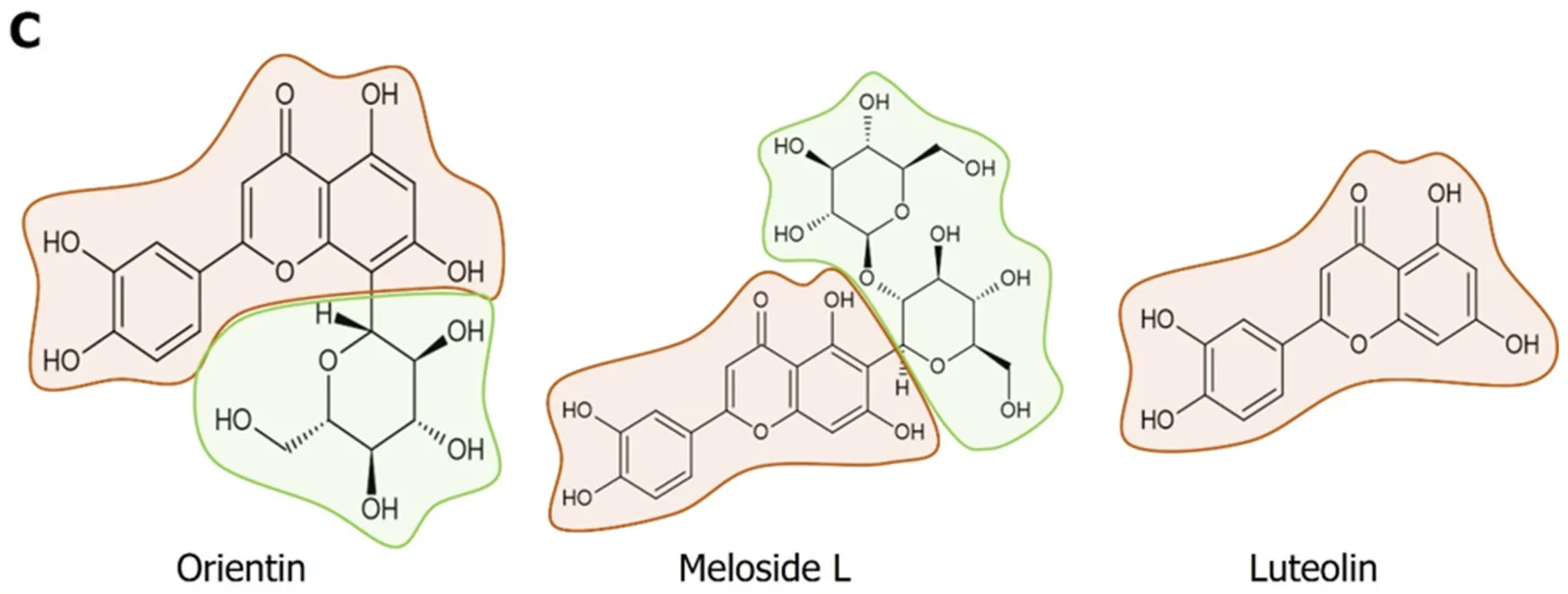
Active compound–target–epilepsy network, functional target profiles, and structural relationships of selected phytochemicals from *P. incarnata*. (**A**) Sankey diagram illustrating the associations among the phytochemical constituents of *P. incarnata*, the top 20 hub genes identified by MCC analysis, and epilepsy. (**B**) Binary compound–target matrix showing predicted associations among targets derived from significantly enriched neuronal GO terms (*p* < 0.05). Targets were grouped into four functional categories: GABAergic neurotransmission (red), monoaminergic neurotransmission (orange), synaptic excitability and neuronal regulation (blue), and carbonic anhydrase/pH regulation (green). Colored squares indicate predicted compound–target associations. (**C**) Chemical structures of orientin, meloside L, and luteolin. The luteolin-related flavone scaffold is highlighted in brown, and the glycosyl moieties of orientin and meloside L are highlighted in green.

**Figure 5:**
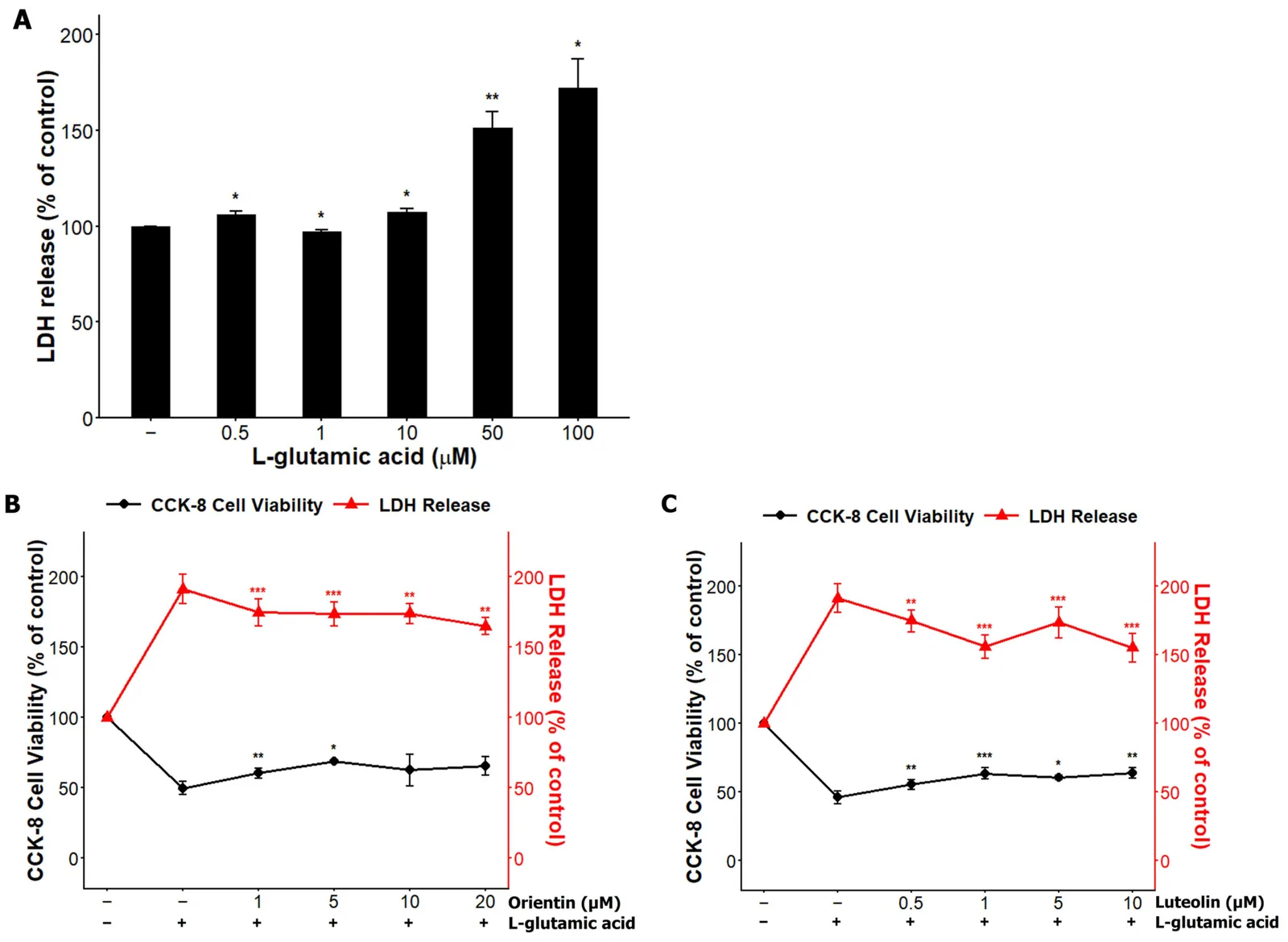
Protective effects of orientin and luteolin against glutamate-induced excitotoxicity in differentiated SH-SY5Y cells. (**A**) LDH release following treatment with various concentrations of L-glutamic acid in the presence of 10 μM glycine. (**B**,**C**) Effects of orientin (**B**) and luteolin (**C**) pretreatment on cell viability (CCK-8) and LDH release following treatment with 100 μM L-glutamic acid and 10 μM glycine. Data are presented as the mean ± SD. * *p* < 0.05, ** *p* < 0.01, and *** *p* < 0.001 vs. control (**A**) or glutamate-treated group (**B**,**C**).

**Figure 6:**
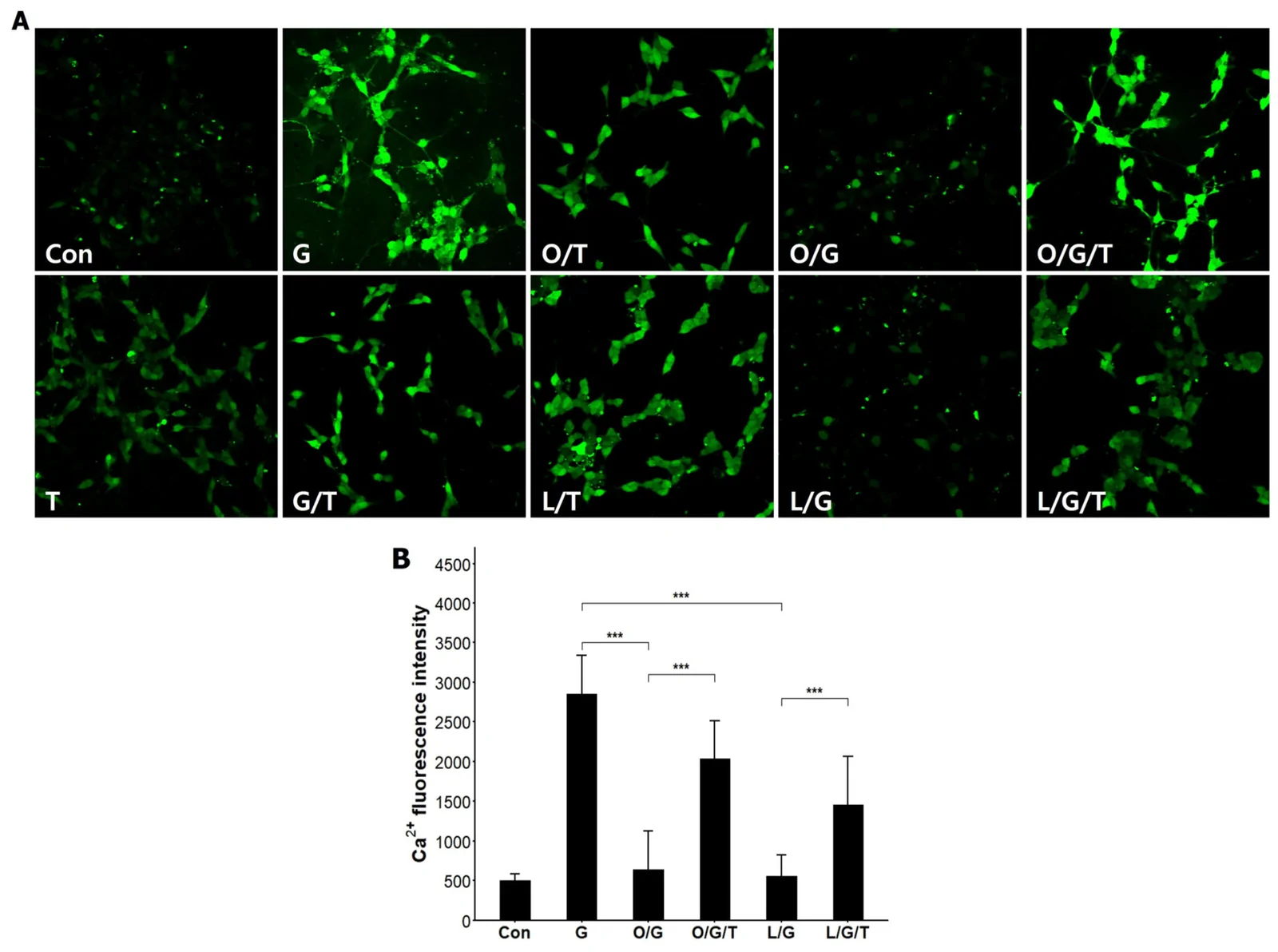
Effects of orientin and luteolin on intracellular Ca^2+^ levels in glutamate-treated SH-SY5Y cells. (**A**) Representative Fluo-4 AM fluorescence images, with intracellular Ca^2+^ -associated fluorescence shown in green. (**B**) Quantification of Fluo-4 fluorescence intensity. Data are presented as the mean ± SD. *** *p* < 0.001 for the indicated comparisons. Con, control; G, 100 μM L-glutamic acid + 10 μM glycine; O, orientin; L, luteolin; T, thapsigargin. Treatment abbreviations are presented in the order of administration.

**Table 1:** Top 20 hub genes in PPI network ranked by MCC score.

Rank	Gene	MCC Score	Rank	Gene	MCC Score
1	CCND1	13,332	11	CCNB1	10,974
2	CYP3A4	12,996	12	CDK2	10,781
3	CYP1A2	12,816	13	IL6	10,337
4	NR1I2	12,606	14	GABRA1	10,264
5	CYP1A1	12,530	15	GABRG2	10,248
6	CYP1B1	11,545	16	GABRA4	10,224
7	ABCG2	11,379	17	GABRA5	10,207
8	CCNA2	11,293	18	GABRA3	10,110
9	CDKN1A	11,264	19	GABRA6	10,104
10	ABCB1	11,146	20	GABRA2	10,082

## Data Availability

The data supporting this study’s findings are available from the corresponding author upon reasonable request.

## Supplementary Materials

The following supporting information can be downloaded at: https://www.mdpi.com/article/10.3390/ijms27198605/s1.

## References

[1] Sills G.J., Rogawski M.A. (2020). Mechanisms of action of currently used antiseizure drugs. Neuropharmacology.

[2] Perucca E., Perucca P., White H.S., Wirrell E.C. (2023). Drug resistance in epilepsy. Lancet Neurol..

[3] Loscher W., Potschka H., Sisodiya S.M., Vezzani A. (2020). Drug Resistance in Epilepsy: Clinical Impact, Potential Mechanisms, and New Innovative Treatment Options. Pharmacol. Rev..

[4] Arenas-Cabrera C., Cabezudo-Garcia P., Calvo-Medina R., Galeano-Bilbao B., Martinez-Agredano P., Ruiz-Gimenez J., Rodriguez-Uranga J.J., Quiroga-Subirana P. (2024). Advances and guidance in the treatment of drug-resistant epilepsy: A review by the Andalusian Epilepsy Society of the new drugs cenobamate, fenfluramine and cannabidiol. Rev. Neurol..

[5] Ng Y.H., Jamil S.N.H., Sarian M.N., Ahmed Q.U., Latip J., Lam S.D., Feroz S.R. (2025). Antiseizure Medications: Advancements, Challenges, and Prospects in Drug Development. Curr. Neuropharmacol..

[6] Rosenfeld W.E. (2025). Epilepsy medication management: Addressing common treatment barriers to adopting cenobamate and other new antiseizure medications. Epilepsia.

[7] Jehi L. (2025). Advances in Therapy for Refractory Epilepsy. Annu. Rev. Med..

[8] Klein P., Friedman D., Kwan P. (2024). Recent Advances in Pharmacologic Treatments of Drug-Resistant Epilepsy: Breakthrough in Sight. CNS Drugs.

[9] De Bellis M., d’Orsi G., Rubino E.M., Arigliano C., Carella M., Sciruicchio V., Liantonio A., De Luca A., Imbrici P. (2025). Adverse effects of antiseizure medications: A review of the impact of pharmacogenetics and drugs interactions in clinical practice. Front. Pharmacol..

[10] Johannessen Landmark C., Eyal S., Burns M.L., Franco V., Johannessen S.I. (2023). Pharmacological aspects of antiseizure medications: From basic mechanisms to clinical considerations of drug interactions and use of therapeutic drug monitoring. Epileptic Disord..

[11] Willems L.M., van der Goten M., von Podewils F., Knake S., Kovac S., Zollner J.P., Rosenow F., Strzelczyk A. (2023). Adverse Event Profiles of Antiseizure Medications and the Impact of Coadministration on Drug Tolerability in Adults with Epilepsy. CNS Drugs.

[12] Brigo F., Marson A. (2022). Approach to the Medical Treatment of Epilepsy. Continuum.

[13] Mesraoua B., Abou-Khalil B., Lattanzi S., Asadi-Pooya A.A., Hail H.A., AbuAlrob M.A., Perucca E. (2026). Novel epilepsy treatments in clinical development. Epilepsy Behav..

[14] Atanasov A.G., Zotchev S.B., Dirsch V.M., International Natural Product Sciences T., Supuran C.T. (2021). Natural products in drug discovery: Advances and opportunities. Nat. Rev. Drug Discov..

[15] Janda K., Wojtkowska K., Jakubczyk K., Antoniewicz J., Skonieczna-Zydecka K. (2020). *Passiflora incarnata* in Neuropsychiatric Disorders-A Systematic Review. Nutrients.

[16] Miroddi M., Calapai G., Navarra M., Minciullo P.L., Gangemi S. (2013). *Passiflora incarnata* L.: Ethnopharmacology, clinical application, safety and evaluation of clinical trials. J. Ethnopharmacol..

[17] Appel K., Rose T., Fiebich B., Kammler T., Hoffmann C., Weiss G. (2011). Modulation of the gamma-aminobutyric acid (GABA) system by *Passiflora incarnata* L.. Phytother. Res..

[18] Elsas S.M., Rossi D.J., Raber J., White G., Seeley C.A., Gregory W.L., Mohr C., Pfankuch T., Soumyanath A. (2010). *Passiflora incarnata* L. (Passionflower) extracts elicit GABA currents in hippocampal neurons in vitro, and show anxiogenic and anticonvulsant effects in vivo, varying with extraction method. Phytomedicine.

[19] Singh B., Singh D., Goel R.K. (2012). Dual protective effect of *Passiflora incarnata* in epilepsy and associated post-ictal depression. J. Ethnopharmacol..

[20] Nassiri-Asl M., Shariati-Rad S., Zamansoltani F. (2007). Anticonvulsant effects of aerial parts of *Passiflora incarnata* extract in mice: Involvement of benzodiazepine and opioid receptors. BMC Complement. Altern. Med..

[21] da Fonseca L.R., Rodrigues R.A., Ramos A.S., da Cruz J.D., Ferreira J.L.P., Silva J.R.A., Amaral A.C.F. (2020). Herbal Medicinal Products from Passiflora for Anxiety: An Unexploited Potential. ScientificWorldJournal.

[22] Noor F., Tahir Ul Qamar M., Ashfaq U.A., Albutti A., Alwashmi A.S.S., Aljasir M.A. (2022). Network Pharmacology Approach for Medicinal Plants: Review and Assessment. Pharmaceuticals.

[23] Jo H.G., Seo J., Jang B., Kim Y., Kim H., Baek E., Park S.Y., Lee D. (2025). Integrating network pharmacology and experimental validation to advance psoriasis treatment: Multi-target mechanistic elucidation of medicinal herbs and natural compounds. Autoimmun. Rev..

[24] Grundmann O., Wang J., McGregor G.P., Butterweck V. (2008). Anxiolytic activity of a phytochemically characterized *Passiflora incarnata* extract is mediated via the GABAergic system. Planta Med..

[25] Soulimani R., Younos C., Jarmouni S., Bousta D., Misslin R., Mortier F. (1997). Behavioural effects of *Passiflora incarnata* L. and its indole alkaloid and flavonoid derivatives and maltol in the mouse. J. Ethnopharmacol..

[26] Ozarowski M., Karpinski T.M. (2021). Extracts and Flavonoids of Passiflora Species as Promising Anti-inflammatory and Antioxidant Substances. Curr. Pharm. Des..

[27] Hopkins A.L. (2008). Network pharmacology: The next paradigm in drug discovery. Nat. Chem. Biol..

[28] Li S., Zhang B. (2013). Traditional Chinese medicine network pharmacology: Theory, methodology and application. Chin. J. Nat. Med..

[29] Zhang P., Zhang D., Zhou W., Wang L., Wang B., Zhang T., Li S. (2023). Network pharmacology: Towards the artificial intelligence-based precision traditional Chinese medicine. Brief. Bioinform..

[30] Panossian A. (2025). Trends and Pitfalls in the Progress of Network Pharmacology Research on Natural Products. Pharmaceuticals.

[31] Wang X., Hu Y., Zhou X., Li S. (2022). Editorial: Network pharmacology and traditional medicine: Setting the new standards by combining In silico and experimental work. Front. Pharmacol..

[32] Arundine M., Tymianski M. (2003). Molecular mechanisms of calcium-dependent neurodegeneration in excitotoxicity. Cell Calcium.

[33] Green J.L., Dos Santos W.F., Fontana A.C.K. (2021). Role of glutamate excitotoxicity and glutamate transporter EAAT2 in epilepsy: Opportunities for novel therapeutics development. Biochem. Pharmacol..

[34] Barker-Haliski M., White H.S. (2015). Glutamatergic Mechanisms Associated with Seizures and Epilepsy. Cold Spring Harb. Perspect. Med..

[35] Chen T.S., Huang T.H., Lai M.C., Huang C.W. (2023). The Role of Glutamate Receptors in Epilepsy. Biomedicines.

[36] Wu W.L., Gong X.X., Qin Z.H., Wang Y. (2025). Molecular mechanisms of excitotoxicity and their relevance to the pathogenesis of neurodegenerative diseases—An update. Acta Pharmacol. Sin..

[37] Dong X.X., Wang Y., Qin Z.H. (2009). Molecular mechanisms of excitotoxicity and their relevance to pathogenesis of neurodegenerative diseases. Acta Pharmacol. Sin..

[38] Zhou X., Chen Z., Xiao L., Zhong Y., Liu Y., Wu J., Tao H. (2022). Intracellular calcium homeostasis and its dysregulation underlying epileptic seizures. Seizure.

[39] Rogawski M.A., Loscher W. (2004). The neurobiology of antiepileptic drugs. Nat. Rev. Neurosci..

[40] Mao X.Y., Zhou H.H., Jin W.L. (2019). Redox-Related Neuronal Death and Crosstalk as Drug Targets: Focus on Epilepsy. Front. Neurosci..

[41] Verma M., Lizama B.N., Chu C.T. (2022). Excitotoxicity, calcium and mitochondria: A triad in synaptic neurodegeneration. Transl. Neurodegener..

[42] Kritis A.A., Stamoula E.G., Paniskaki K.A., Vavilis T.D. (2015). Researching glutamate-induced cytotoxicity in different cell lines: A comparative/collective analysis/study. Front. Cell. Neurosci..

[43] Mendez-Armenta M., Nava-Ruiz C., Juarez-Rebollar D., Rodriguez-Martinez E., Gomez P.Y. (2014). Oxidative stress associated with neuronal apoptosis in experimental models of epilepsy. Oxid. Med. Cell. Longev..

[44] Feng Y., Chen S., Zhao Y., Wu D., Li G. (2022). Heterocyclic aromatic amines induce Neuro-2a cells cytotoxicity through oxidative stress-mediated mitochondria-dependent apoptotic signals. Food Chem. Toxicol..

[45] Stocco M.R., Tolledo C., Wadji F.B., Gonzalez F.J., Miksys S., Tyndale R.F. (2020). Human CYP2D6 in the Brain Is Protective Against Harmine-Induced Neurotoxicity: Evidence from Humanized CYP2D6 Transgenic Mice. Mol. Neurobiol..

[46] Vongthip W., Nilkhet S., Boonruang K., Sukprasansap M., Tencomnao T., Baek S.J. (2024). Neuroprotective mechanisms of luteolin in glutamate-induced oxidative stress and autophagy-mediated neuronal cell death. Sci. Rep..

[47] Tian T., Zeng J., Zhao G., Zhao W., Gao S., Liu L. (2018). Neuroprotective effects of orientin on oxygen-glucose deprivation/reperfusion-induced cell injury in primary culture of rat cortical neurons. Exp. Biol. Med..

[48] Johnson J.W., Ascher P. (1987). Glycine potentiates the NMDA response in cultured mouse brain neurons. Nature.

[49] Cheng C., Fass D.M., Reynolds I.J. (1999). Emergence of excitotoxicity in cultured forebrain neurons coincides with larger glutamate-stimulated [Ca^2+^]_i_ increases and NMDA receptor mRNA levels. Brain Res..

