# Supplementary material for: Network Pharmacology-Guided Identification of Orientin from Passiflora incarnata Reveals Neuroprotective Effects Against Glutamate-Induced Excitotoxicity Through Regulation of Calcium Homeostasis
Source: Int J Mol Sci. 2026 Sep 25;27(19):8605. doi: 10.3390/ijms27198605 (PMC13665118; doi:10.3390/ijms27198605)
Supplement: Supplementary file 1 [file ijms-27-08605-s001.zip › ijms-4548008-supplementary.pdf]

Network Pharmacology-Guided Identification of Orientin from Passiflora incarnata Reveals Neuroprotective Effects Against Glutamate-Induced Excitotoxicity Through Regulation of Calcium Homeostasis

Jinyoung Park [1], Muhammad Yasir [1], Eun-Taek Han [2], Jin-Hee Han [2], Won Sun Park [3], Jongseon Choe [4] and Wanjoo Chun [1,*]

[1] Department of Pharmacology, Kangwon National University School of Medicine, Chuncheon 24341, Republic of Korea; jinyoung0326@kangwon.ac.kr (J.P.); yasir.khokhar1999@gmail.com (M.Y.)
[2] Department of Medical Environmental Biology and Tropical Medicine, Kangwon National University School of Medicine, Chuncheon 24341, Republic of Korea; ethan@kangwon.ac.kr (E.-T.H.); han.han@kangwon.ac.kr (J.-H.H.)
[3] Department of Physiology, Kangwon National University School of Medicine, Chuncheon 24341, Republic of Korea; parkws@kangwon.ac.kr
[4] Department of Microbiology and Immunology, Kangwon National University School of Medicine, Chuncheon 24341, Republic of Korea; jchoe@kangwon.ac.kr
* Correspondence: wchun@kangwon.ac.kr; Tel.: +82-33-250-8853

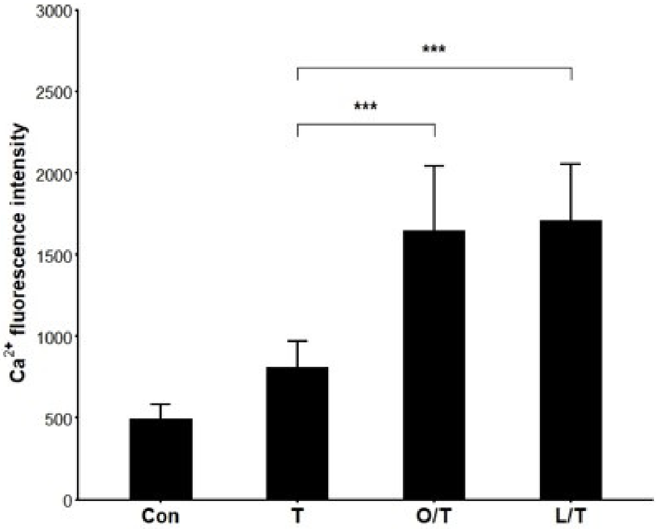

Figure S1. Effects of orientin and luteolin on thapsigargin-induced intracellular Ca^{2+} responses in the absence of glutamate. Differentiated SH-SY5Y cells were pretreated with orientin or luteolin in the absence of glutamate, followed by treatment with thapsigargin. Fluo-4 fluorescence intensity was quantified as an indicator of intracellular Ca^{2+} levels. Groups were designated as thapsigargin alone (T), orientin followed by thapsigargin (O/T), and luteolin followed by thapsigargin (L/T). Data are presented as the mean ± SD. *** $p < 0.001$ vs. T.
